# Designing a species-selective lure based on microbial volatiles to target *Lobesia botrana*

**DOI:** 10.1038/s41598-020-63088-3

**Published:** 2020-04-16

**Authors:** Sebastian Larsson Herrera, Péter Rikk, Gabriella Köblös, Magdolna Olívia Szelényi, Béla Péter Molnár, Teun Dekker, Marco Tasin

**Affiliations:** 1 SLU, Department of Plant Protection Biology, 230 53 Alnarp, Sweden; 2 Plant Protection Institute, Centre for Agricultural Research, Budapest, Hungary

**Keywords:** Microbial ecology, Plant ecology, Biodiversity, Invasive species

## Abstract

Sustainable, low impact control methods, including mating disruption and microbial insecticides against *L. botrana* have been available for decades. Yet, successful implementation has been restricted to only a few grapevine districts in the world. A limiting factor is the lack of a female attractant to either monitor or control the damaging sex. Volatile attractants for both female and male insects can be used to assess when *L. botrana* populations exceed economic thresholds, and to decrease the use of synthetic pesticides within both conventional and pheromone programs. Rather than using host-plant volatiles, which are readily masked by background volatiles released by the main crop, we tested the attractiveness of volatiles that signify microbial breakdown and more likely stand out against the background odour. A two-component blend of 2-phenylethanol (2-PET) and acetic acid (AA) caught significant numbers of both sexes. Catches increased with AA and, to a minimal extent, 2-PET loads. However, a higher load of 2-PET also increased bycatches, especially of Lepidoptera and Neuroptera. Major (ethanol, ethyl acetate, 3-methyl-1-butanol) or minor (esters, aldehydes, alcohols and a ketone) fermentation volatiles, did surprisingly not improve the attraction of *L. botrana* compared to the binary blend of 2-PET and AA alone, but strongly increased bycatches. The most attractive lure may thus not be the best choice in terms of specificity. We suggest that future research papers always disclose all bycatches to permit evaluation of lures in terms of sustainability.

## Introduction

The replacement of synthetic pesticides with selective, low-impact innovations is an important prerequisite to develop more sustainable agricultural production systems at the landscape level^1,2^. The challenge is particularly significant in cultivated monocultures such as orchards and vineyards, which represent generous ‘invitations’ to pests, while disfavoring natural control mechanisms^3^.

In vineyards, the grapevine moth *Lobesia botrana* (Denis & Schiffermüller) is among the most important pests and requires regular insecticide applications^4^. Although the technology of mating disruption has been available for *L. botrana* for almost three decades, implementation is only achieved on a restricted number of viticultural districts in the world^5^. Factors that limited the spread of this environmentally friendly technology are among others, the challenge to involve a critical number of motivated stakeholders to reach an area-wide approach, and the lack of reliable attractants to monitor pest populations within a pheromone permeated crop^6^. Similarly, the use of microbial agents with a lower consistency than conventional insecticides requires meticulous monitoring to assess the efficacy, and thus are adopted only by either motivated growers or wine districts with advanced extension services^7^.

Availability of a monitoring tool to forewarn growers and advisors when the population of the grapevine moth exceeds damage threshold would facilitate the implementation of both mating disruption and biocontrol application. Whereas effective monitoring tools are already identified for several other tortricid pests^8–10^, further investigations are needed in *L. botrana*. Previous studies showed attraction of both sexes of *L. botrana* to volatiles emitted by host plants, including grapevine *Vitis vinifera* and flax-leaved daphne *Daphne gnidium*^11–13^. Although promising, these laboratory and semi-field results were not mirrored by trap catches in the field, due possibly to a suboptimal release of single compounds and blend ratios from dispensers, suboptimal trap properties, and the competition with the background volatiles emitted by the crop^14^.

The issue of host plant background odor masking the lure may be circumvented by instead using volatiles that stand out against the background odors, such as volatiles associated with microbial breakdown^15^. Recently, microbial volatiles identified from grapes were screened in behavioural experiments in South American vineyards and a blend of two microbial compounds, acetic acid (AA) and 2-phenylethanol (2-PET), was identified as attractive for both sexes of *L. botrana*^16^. Whereas the field attraction of this two component blend was further corroborated by El Sayed *et al*. 2019^17^, the importance of the component ratio in the same blend remains, to the best of our knowledge, to be investigated. We hypothesized that a ratio skewed toward AA would increase the trap attraction range for the grapevine moth, while a 1:1 ratio would instead decrease the lure specificity without augmenting *L. botrana* catches. To test this hypothesis, we measured field attraction towards traps baited with six different loads of AA/2-PET (5:500, 50:500, 500:500, 500:50, 500:5 and 50:50). Beside testing for the first time the importance of ratio and load of these two components in conventionally managed European vineyards, we also investigated the significance of additional microbial compounds to further enhance attraction. Because *L. botrana* responded to volatiles released by grapes inoculated with microorganisms such as yeasts (*Hanseniaspora uvarum, Metschnikowia pulcherrima, Pichia anomala*, *Saccharomyces cerevisiae*) or sour rot bacteria (*Acetobacter aceti*, *Gluconobacter oxydans*)^16^, we hypothesized that a more complete blend mimicking microbial release would enhance trap catches in comparison to the reference two-component blend. Finally, we evaluated the selectivity of the lure, a hallmark of sustainable pest control innovation, by carefully analysing catches of non-target species.

## Material and Methods

### Vineyards

Trapping tests were carried out during 2018 in two commercial vineyards in the Eger wine region in North-Eastern Hungary in the municipality of Maklár. Vineyards (6 and 7 hectares, respectively) were planted at a density of 4000 vine ha^−1^. Grapevine plants were planted at 2.5 × 1 m and belonged to the variety ‘Merlot’, ‘Kékfrankos’, ‘Turán’, ‘Cabernet franc’. An integrated pest management program^18^ was applied all along the season to control pests and diseases. To control *L. botrana*, Avaunt (Indoxacarb, 150 g/l) and Actara SC (Thiametoxam, 240 g/l) were applied on May 17 and on July 14, respectively. Although sprayed with insecticides, we selected these fields due to the very high pest population reported in the previous season.

### Volatile compounds

Major microbial volatiles emanating from inoculated grapes^16^ were tested on their attractiveness for *L. botrana*. These were added to an existing 2-component blend consisting of AA and 2-PET. Microbial volatiles were formulated in polyethylene Eppendorf vials^16^. Synthetic volatiles included acetic acid (AA, 99.8%; VWR Chemicals, Belgium), 2-phenylethanol (2-PET, 99%; Acros Organics, China), ethanol (96%; VWR Chemicals, France), 3-methyl-1-butanol (99%; Acros Organics, Germany), ethyl acetate (99.5%; Riedel-de Haën, Germany), isobutanol (99.75%; Fisher Chemical, England), 3-methyl-3-buten-1-ol (97%; Acros Organics, Germany), isoamyl acetate (99.5%; Fisher Chemical, England), isobutyl acetate (98%; Acros Organics, Germany), methyl acetate (99%; Acros Organics, Belgium), acetaldehyde (99%; Fisher Chemical, England), benzaldehyde (99.5%; Sigma-Aldrich, USA), 3-hydroxy-2-butanone (acetoin) (95%; Sigma-Aldrich, China). Except for AA and 2-PET, all other chemicals were pipetted into the vial onto a dental cotton plug as neat compounds at 100 mg each. To test whether or not blending AA and 2-PET in a single vial would affect moth attraction, AA/2-PET field performance was evaluated with the two compounds loaded either in the same or in two different vials (S and D in Tables 1–3). In order to more evenly release the compounds and over a longer time, 100 mg of paraffin was added onto the cotton plug (see Tables 1–3 for description of attractants). Vials were hung at the centre of a transparent plastic delta trap with a replaceable sticky insert of 160 ×100 mm (Csalomon, Budapest, Hungary). Along the rows in the vineyard(s), traps were placed in randomized lines, with 4 rows of vine (12.5 m) between each trap line and 20 m between traps. Traps were inspected two or three times per week and inserts with captures were stored at +5 °C for later identification using a stereomicroscope. Trapping experiments were carried out in 2018 during May 3–17 (first generation), June 14-July 5 (second generation) and August 3–22 (third generation). Pheromone traps loaded with 0.3 mg of E7,Z9-12:Ac (Csalomon, Budapest, Hungary) were installed in an adjacent plot to monitor seasonal activity of males of the pest.

**Table 1:** Target and non-target insect species caught in traps during the first flight (May 3–17, 2018).

Compound		Chemical class					A1	A2	A3	A4	A5	A6	A7	A8	A9	A10	A11	A12	A13	A14	A15
Vial for AA and 2-PET (D = different, S = Same)							D	S	D	D	D	D	D	D	D	D	D	—	S	S	—
acetic acid (AA)		acid					500	500	500	500	500	500	500	500	500	500	500	—	500	500	—
2-phenylethanol (2-PET)		benzene and subs. der.					50	50	50	50	50	50	50	50	50	50	50	—	50	50	—
ethanol		alcohol					—	—	100	100	—	100	—	—	—	—	—	100	—	—	—
3-methyl-1-butanol		alcohol					—	—	100	100	—	—	100	—	—	—	—	100	100	—	—
ethyl acetate		ester					—	—	100	100	—	—	—	100	—	—	—	100	—	—	—
isoamyl acetate		ester					—	—	100	—	—	—	—	—	—	100	—	—	—	—	—
isobutyl acetate		ester					—	—	100	—	—	—	—	—	—	100	—	—	—	—	—
methyl acetate		ester					—	—	100	—	—	—	—	—	—	100	—	—	—	—	—
isobutanol		alcohol					—	—	100	—	—	—	—	—	100	—	—	—	—	100	—
3-methyl-3-buten-1-ol		alcohol					—	—	100	—	—	—	—	—	100	—	—	—	—	100	—
acetaldehyde		aldehyde					—	—	100	—	—	—	—	—	—	—	100	—	—	—	—
benzaldehyde		aldehyde					—	—	100	—	—	—	—	—	—	—	100	—	—	—	—
acetoin		acyloins					—	—	—	—	100	—	—	—	—	—	—	—	—		—
**Order**	**Family**	**Species**	**Stat**	**p-val**	χ**2**	**P** χ	**A1**	**A2**	**A3**	**A4**	**A5**	**A6**	**A7**	**A8**	**A9**	**A10**	**A11**	**A12**	**A13**	**A14**	**A15**
Lepidoptera	Tortricidae	*Lobesia botrana* (female)	P	0.927	18.9	0.927	5 a	10 a	9 a	2 a	5 a	4 a	6 a	1 a	6 a	1 a	—	—	—	—	—
Lepidoptera	Tortricidae	*Lobesia botrana* (male)	P	0.588	12.4	0.588	1 a	6 a	5 a	4 a	6 a	3 a	2 a	1 a	4 a	2 a	3 a	—	1 a	—	—
Lepidoptera	Tortricidae	*Lobesia botrana* (total)	P	0.204	36.2	0.204	6 a	16 a	14 a	6 a	11 a	7 a	8 a	2 a	10 a	3 a	3 a	—	1 a	—	—
Coleoptera	Coccinellidae	*Coccinellidae*	—				—	1	—	—	2	—	—	—	1	—	—	—	—	—	—
Diptera	Muscidae	*Musca spp.*	NB	0.000	333.1	0.000	11 ab	6 a	175 ef	122 def	20 ab	77 ce	10 ab	40 bc	174 ef	20 ab	5 a	18 ac	8 acd	117 f	6 a
Diptera	Syrphidae	Syrphidae	—				—	—	—	—	—	1	—	—	—	—	—	—	—	—	—
Hemiptera	Auchenrorrchyncha (suborder)	Auchenrorrchyncha	—				—	—	—	—	—	—	—	—	—	1	—	—	—	—	—
Lepidoptera	Geometridae	*Ematurga atomaria*	—				—	—	2	1	—	—	1	—	—	—	1	—	3	—	—
Lepidoptera	Noctuidae	*Acronicta psi*	—				—	—	—	—	—	—	—	—	—	—	—	—	1	—	—
Lepidoptera	Noctuidae	*Agrotis exclamationis*	P	0.168	6.7	0.168	—	—	7 a	3 a	—	1 a	4 a	—	2 a	—	—	—	3 a	2 a	—
Lepidoptera	Noctuidae	*Dypterygia scabriuscula*	P	0.220	24.4	0.22	2 a	1 a	7 a	4 a	—	1 a	11 a	—	5 a	4 a	—	1 a	3 a	1 a	—
Lepidoptera	Noctuidae	*Dysgonia algira*	—				—	—	3	1	—	—	—	—	—	—	—	1	—	—	—
Lepidoptera	Noctuidae	*Lacanobia oleracea*	P	0.242	9.1	0.242	—	—	—	1 a	—	1 a	7 a	—	3 a	1 a	—	—	2 a	—	—
Lepidoptera	Noctuidae	*Mythimna albipuncta*	P	0.830	8.0	0.83	—	—	7 a	8 a	1 a	—	8 a	—	—	5 a	—	—	6 a	5 a	—
Lepidoptera	Noctuidae	Noctuidae	P	0.621	2.2	0.621	1 a	1 a	2 a	3 a	—	—	1 a	—	2 a	1 a	—	—	—	—	—
Lepidoptera	Noctuidae	*Trachea atriplicis*	—				—	—	—	—	—	—	1	—	1	—	—	—	—	1	—
Lepidoptera	Nymphalidae	*Apatura irisis*	—				—	—	—	—	—	—	1	—	—	1	—	—	—	—	—
Lepidoptera	Pyralidae	*Hypsopygia costalis*	P	0.862	27.6	0.862	—	—	3 a	18 b	—	—	10 ab	—	3 a	1 a	—	—	5 ab	—	—
Lepidoptera	Pyralidae	Pyralidae	—				—	—	1	1	—	—	1	—	2	—	—	—	1	2	—
Lepidoptera	Pyralidae	*Pyralis farinalis*	—				—	—	—	1	—	—	3	—	—	—	—	—	1	—	—
Lepidoptera	Sphingidae	*Deilephila porcellus*	—				—	—	1	—	—	—	—	—	—	—	—	—	—	—	—
Lepidoptera	Thyatiridae	*Habrosyne pyriotides*	—				—	—	—	3	—	—	—	—	—	—	—	—	—	—	—
Lepidoptera	Thyatiridae	*Tethea ocularis*	—				—	—	1	—	—	—	1	—	—	—	—	—	—	—	—
Lepidoptera	Thyatiridae	*Thyatira batis*	—				—	—	—	1	—	—	—	—	—	—	—	—	—	1	—
Lepidoptera	Thyatiridae	Thyatiridae	—				—	—	2	—	—	1	—	—	—	—	—	—	—	—	—
Lepidoptera	Tortricidae	*Hedya pruniana*	P	0.775	53.2	0.775	2 a	3 a	20 a	9 a	2 a	2 a	10 a	2 a	2 a	3 a	3 a	—	2 a	—	1 a
Lepidoptera	Tortricidae	*Olethreutes arcuella*	—				—	—	—	2	—	—	—	—	—	—	—	—	—	—	—
Lepidoptera	Tortricidae	*Ptycholoma lecheana*	—				1	—	—	1	1	—	—	2	—	—	1	—	—	—	—
Lepidoptera	Tortricidae	*Tortrix viridana*	—				—	—	1	—	1	—	—	1	—	—	—	—	—	—	—
Neuroptera	Chrysopidae	*Chrysoperla spp*	—				1	—	—	—	—	—	—	1	—	—	—	—	—	—	—

Tested blends: A1-A15. Stat: Poisson (P) or negative binomial (NB) distribution. P-val: probability value for overdispersion with poisson distribution. Σ2: chi-square value for factor treatment, PΣ: probability for the differences between treatments.

**Table 2:** Target and non-target insect species caught during the second flight (June 14 - July 5 2018).

Compound		Chemical class					B1	B2	B3	B4	B5	B6	B7
Vial for AA and 2-PET (D = different, S = Same)							D	S	S	S	S	S	—
acetic acid (AA)		acid					500	500	500	500	500	500	—
2-phenylethanol (2-PET)		benzene and subs. der.					50	50	50	50	50	—	
ethanol		alcohol					—	—	100	100	—	—	—
3-methyl-1-butanol		alcohol					—	—	100	100	—	—	—
ethyl acetate		ester					—	—	100	100	—	—	—
isoamyl acetate		ester					—	—	100	—	—	—	
isobutyl acetate		ester					—	—	100	—	—	—	—
methyl acetate		ester					—	—	100	—	—	—	—
isobutanol		alcohol					—	—	100	—	100	—	—
3-methyl-3-buten-1-ol		alcohol					—	—	100	—	100	—	—
acetaldehyde		aldehyde					—	—	100	—	—	—	—
benzaldehyde		aldehyde					—	—	100	—	—	—	—
acetoin		acyloins					—	—	100	—	—	100	—
**Order**	**Family**	**Species**	**Stat**	**P-val**	**χ**^**2**^	**P χ**	**B1**	**B2**	**B3**	**B4**	**B5**	**B6**	**B7**
Lepidoptera	Tortricidae	*Lobesia botrana* (female)	NB	0.001	13.9	0.016	40 a	18 a	43 a	26 a	17 a	19 a	—
Lepidoptera	Tortricidae	*Lobesia botrana* (male)	P	0.895	37.6	0.000	25 b	11 ab	24 b	9 ab	11 ab	17 ab	1 a
Lepidoptera	Tortricidae	*Lobesia botrana* (total)	NB	0.000	71.2	0.000	65 b	29 b	67 b	35 b	28 b	36 b	1 a
Coleoptera	Coccinellidae	*Harmonia axyridis*	—				1	—	—	—	—	—	—
Diptera	Culicidae	Culicidae	—				—	—	—	—	—	—	1
Diptera	Muscidae	*Musca* spp.	NB	0.000	85.1	0.000	—	2 a	36 bc	11 ab	108 c	4 a	1 a
Hemiptera	Auchenrorrchyncha (suborder)	Auchenrorrchyncha	P	0.918	3.9	0.562	3 a	5 a	—	2 a	1 a	2 a	4 a
Hymenoptera	Apoidea (old family)	Apoidea	—				—	—	—	1	—	—	—
Hymenoptera	Vespidae	Vespidae	—				1	—	—	—	—	1	—
Lepidoptera	Noctuidae	*Autographa gamma*	—				1	—	—	—	—	—	—
Lepidoptera	Papilionidae	Papilionidae	—				—	—	1	—	3	—	—
Lepidoptera	Pyralidae	Pyralidae	—				1	—	—	—	—	—	—
Lepidoptera	Tortricidae	Tortricidae	P	0.120	18.7	0.002	5 a	2 a	13 a	1 a	—	2 a	3 a
Neuroptera	Chrysopidae	*Chrysoperla* spp.	NB	0.026	9.6	0.142	10 a	12 a	6 a	5 a	4 a	4 a	1 a

Tested blends: B1-B7. Stat: Poisson (P) or negative binomial (NB) distribution. P-val: probability value for overdispersion with poisson distribution. Σ2: chi-square value for factor treatment, PΣ: probability for the differences between treatments.

**Table 3:** Target and non-target insect species caught in traps during the third flight (August 3–22, 2018).

Compound		Chemical class					C1	C2	C3	C4	C5	C6	C7	C8	C9	C10	C11	C12
Vial for AA and 2-PET (D = different, S = Same)							D	S	S	S	S	S	S	S	S	S	S	—
acetic acid (AA)		acid					500	500	500	500	500	500	500	500	50	5	50	—
2-phenylethanol (2-PET)		benzene and subs. der.					50	50	50	50	50	50	5	500	50	500	500	—
ethanol		alcohol					—	—	100	100	—	—	—	—	—	—	—	—
3-methyl-1-butanol		alcohol					—	—	100	100	—	—	—	—	—	—	—	—
ethyl acetate		ester					—	—	100	100	—	—	—	—	—	—	—	—
isoamyl acetate		ester					—	—	100	—	100	—	—	—	—	—	—	—
isobutyl acetate		ester					—	—	100	—	100	—	—	—	—	—	—	—
methyl acetate		ester					—	—	100	—	100	—	—	—	—	—	—	—
isobutanol		alcohol					—	—	100	—	—	—	—	—	—	—	—	—
3-methyl-3-buten-1-ol		alcohol					—	—	100	—	—	—	—	—	—	—	—	—
acetaldehyde		aldehyde					—	—	100	—	—	100	—	—	—	—	—	—
benzaldehyde		aldehyde					—	—	100	—	—	100	—	—	—	—	—	—
acetoin		acyloins					—	—	100	—	—	—	—	—	—	—	—	—
Order	**Family**	**Species**	**Stat**	**p-val**	χ**2**	**P** χ	**C1**	**C2**	**C3**	**C4**	**C5**	**C6**	**C7**	**C8**	**C9**	**C10**	**C11**	**C12**
Lepidoptera	Tortricidae	*Lobesia botrana* (female)	NB	0.000	142.6	0.000	129 c	121 c	160 c	81 bc	96 c	104 c	117 c	154 c	86 c	33 b	75 bc	2 a
Lepidoptera	Tortricidae	*Lobesia botrana* (male)	NB	0.000	136	0.000	290 cd	285 cd	383 d	179 bc	271 cd	216 bd	321 cd	298 cd	211 bd	112 b	172 bc	25 a
Lepidoptera	Tortricidae	*Lobesia botrana* (total)	NB	0.000	170.5	0.000	419 cd	406 cd	543 d	260 bc	367 cd	320 cd	438 cd	452 cd	297 bd	145 b	247 bc	27 a
Coleoptera	Coccinellidae	Coccinellidae	—				1	—	1	1	2	1	1	—	1	—	1	1
Coleoptera	Coccinellidae	*Harmonia axyridis*	—				—	—	—	—	—	—	—	—	—	1	—	—
Diptera	Drosophilidae	*Drosophila* spp.	P	0.053	18.3	0.000	—	—	68 b	27 a	—	—	—	—	—	—	—	—
Diptera	Muscidae	*Musca* spp.	P	0.060	124.9	0.000	2 a	4 a	27 b	39 b	3 a	3 a	3 a	—	—	2 a	1 a	—
Hemiptera	Flatidae	Flatidae	P	0.184	7	0.800	7 a	5 a	7 a	5 a	2 a	6 a	8 a	3 a	6 a	7 a	7 a	5 a
Hymenoptera	Vespidae	Vespidae	—				2	—	1	1	—	—	—	1	—	1	—	1
Lepidoptera	Drepanidae	*Habrosyne pyriotides*	—				—	—	—	1	—	—	—	—	—	—	—	—
Lepidoptera	Erebidae	*Grammodes geometrica*	—				—	—	—	—	1	—	—	—	—	—	—	—
Lepidoptera	Noctuidae	*Agrotis exclamationis*	—				—	—	1	2	—	—	—	1	—	1	—	—
Lepidoptera	Noctuidae	*Autographa gamma*	—				—	—	—	—	—	—	—	1	—	1	—	—
Lepidoptera	Noctuidae	*Dypterygia scabriuscula*	P	0.998	3.8	0.875	2 a	5 a	1 a	2 a	3 a	—	2 a	3 a	3 a	—	2 a	—
Lepidoptera	Noctuidae	*Mythimna albipuncta*	—				—	—	1	1	1	—	—	—	—	—	—	—
Lepidoptera	Noctuidae	*Trachea atriplicis*	—				—	—	1	1	—	—	—	—	—	—	—	—
Lepidoptera	Pyralidae	*Hypsopygia costalis*	P	0.058	126.2	0.000	—	1 a	48 b	55 b	—	—	—	—	1 a	—	—	—
Lepidoptera	Pyralidae	*Pyralis farinalis*	—				—	—	1	—	—	—	—	—	—	—	—	—
Lepidoptera	Tortricidae	*Pandemis* spp.	—				—	1	—	—	1	2	—	—	—	—	—	—
Lepidoptera	Tortricidae	Tortricidae	—				—	—	1	3	—	—	—	—	—	1	—	—
Neuroptera	Chrysopidae	*Chrysoperla* spp.	P	0.123	80.7	0.000	4 ab	2 b	7 ab	—	2 b	3 b	—	29 c	10 bc	16 bc	21 ac	1 ab

Tested blends: C1-C12. Stat: Poisson (P) or negative binomial (NB) distribution. P-val: probability value for overdispersion with poisson distribution. Σ2: chi-square value for factor treatment, PΣ: probability for the differences between treatments.

### Statistical analysis

R was used for statistical analyses and visualisations^19^. A function was developed using the ‘tidyverse’^20^ to analyze the catches of target and non target species using the following workflow and criterias; (1) If less than 10 insects were caught across all treatments, no stats was performed, (2) If the number of insects caught for a species was less than 100 in each flight period, the catches were pooled across dates, (3) for species with more than 100 catches, dates with no insect of a given species in any of the treatments were filtered out. Data was subsequently fitted to a Poisson generalized linear model (glm) and tested for overdispersion using the package AER^21^. If the data were significantly overdispersed (p < 0.05), the Poisson model was replaced by the correspondent negative binomial, setting the maximum likelihood “theta” as extracted with library MASS^22^. Treatments in the model were compared pairwise using the package multcomp^23^. Treatments with no catches were omitted from the analysis. Specificity was calculated as the number of catches of target species divided by the total number of catches.

## Results

### *Lobesiabotrana* captures

**Figure 1:**
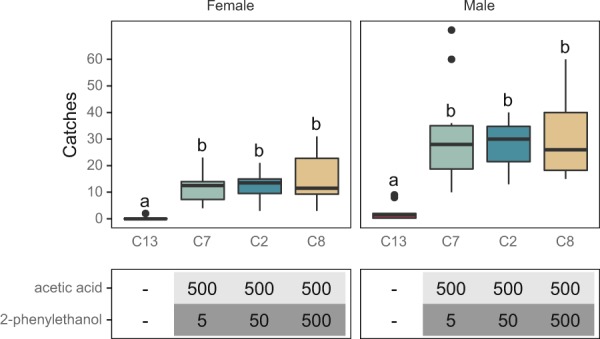
Comparison of capture rates of *L. botrana* males and females with a 2-component lure with an increasing load (mg) of 2-PET. Experiments were carried out in 2018 (August 2–22). Bars with different letters differ significantly. A total of 394 females and 929 males were caught.

**Figure 2:**
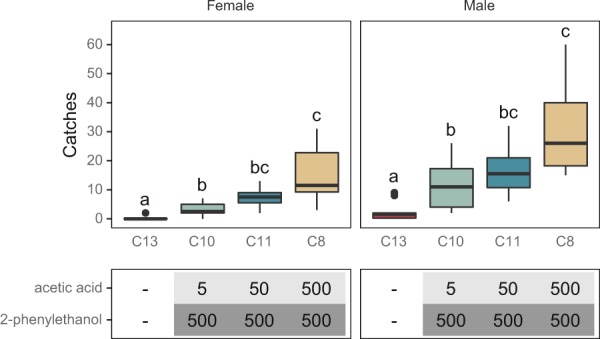
Comparison of capture rates of *L. botrana* males and females with a 2-component lure with an increasing load (mg) of AA. Experiments were carried out in 2018 (August 2–22). Bars with different letters differ significantly. A total of 264 females and 607 males were caught.

**Figure 3:**
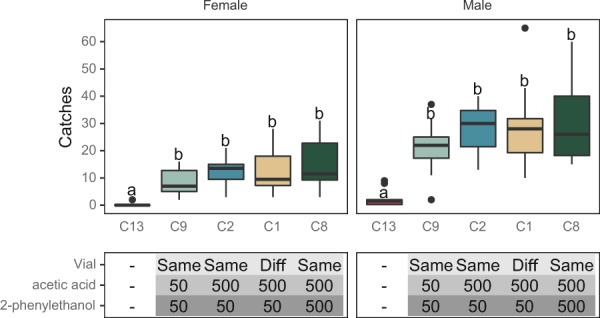
Boxplot of capture rates of *L. botrana* male and female in traps baited with AA and 2-PET (mg) loaded in the same (Same) or in two different (Diff) vials. Experiments were carried out in 2018 (August 2–22). Bars with different letters differ significantly. A total of 492 females and 1109 males were caught.

**Figure 4:**
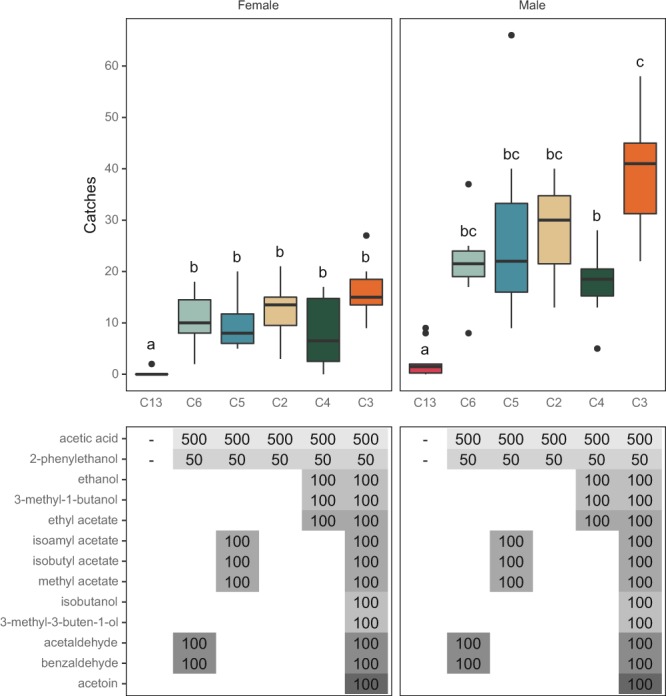
Boxplot of capture rates of *L. botrana* males and females in traps baited with different blends of microbial volatiles (mg). Experiments were carried out in 2018 (August 2–22). Bars with different letters differ significantly. A total of 564 females and 1359 males were caught.

**Figure 5:**
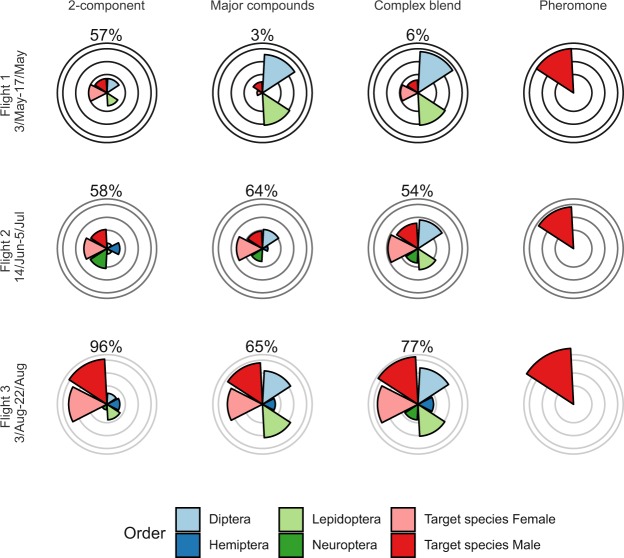
Total captures (square root transformed) of *Lobesia botrana* (target species) and other insect orders during three flight periods within the same fields. The flight period is presented on the y-axis. Concentric lines indicate 10, 50, 250 and 500 insects caught. The percentage of target species caught is indicated at the top of each radial plot. The composition of each of the three blends can be found in Fig. 4 (last three bars) and Table 3. The 2-component lure consisted of 500 mg AA and 50 mg 2-PET loaded in the same vial. The radial plots furthest to the right represents the number of *L. botrana* males caught in sex-pheromone traps during the three flight periods. The comparison of male catches between sex-pheromone and kairomone traps should be done with caution, because the two types of traps were placed within neighbouring plots to avoid interference with each other.

In the first flight a total of 49 females and 38 males were captured in 140 traps. Captures across the 15 treatments are summarized in Table 1. Due to the low population level, no differences between treatments were found. In the second generation a total of 163 female and 98 male *L. botrana* were caught (Table 2). Similarly to the first flight, catches were too low to permit comparison among treatments. In the third generation (Figs. 1–4, Table 3) a much higher population level was present and a total of 1158 females and 2763 males were caught. On average 12.1 females and 28.5 males per trap were caught in traps baited with 500 mg AA and 50 mg 2-PET. Changing the load of 2-PET to 500 or 5 did not affect trap catches of either sex (Fig. 1). However, a 100-fold reduction of the AA load halved the catch of *L. botrana* compared to 500:500 (Fig. 2). A similar ratio of males vs females were caught in all traps baited with any AA:2-PET load (Fig. 3). The number of captures did not differ when AA and 2-PET were loaded within the same or in two separate vials (Fig. 3). Male captures in sex-pheromone traps (275 males/trap) exceeded those of AA:2-PET treatments. Because sex-pheromone traps were placed in a field nearby the one where microbial volatiles were tested, the number of caught males cannot directly be correlated to the catches of the microbial lures. However, it represents an estimation of the population level (Fig. 5).

### Addition of major microbial compounds

The addition of major fermentation compounds released from inoculated grapes (see materials and methods and^16^) including ethanol, 3-methyl-1-butanol and ethyl acetate at 100 mg each did not improve female attraction to the two-component blend of 500 mg AA and 50 mg 2-PET (Fig. 4). Esters (isoamyl acetate, isobutyl acetate, methyl acetate), aldehydes (acetaldehyde, benzaldehyde) or acetoin added to the two-component blend of AA and 2-PET did not improve catches of either sex compared to the two-component blend (Fig. 4). A lower number of males was captured by the 5-component in comparison with the 13-component blend (Fig. 4).

### Bycatches

**Figure 6:**
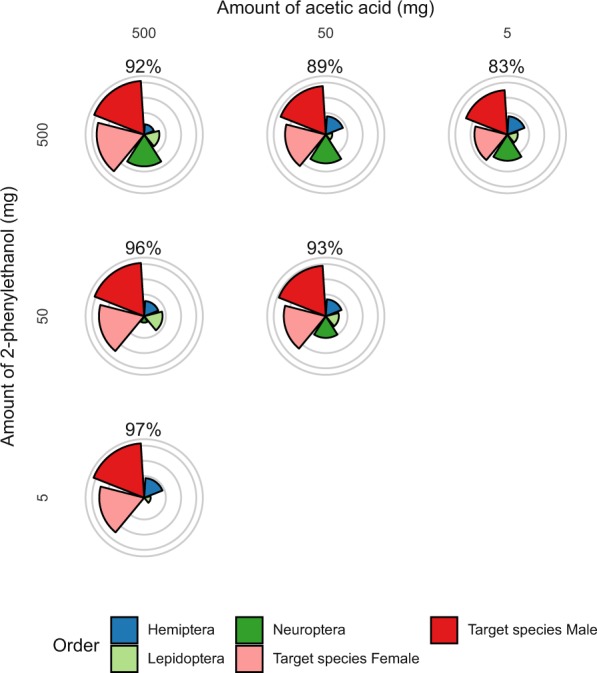
Total captures (square root transformed) of *Lobesia botrana* (target species) and other species during the third flight in traps baited with a different load of AA and 2-PET. Concentric lines indicate 10, 50, 250 and 500 insects caught. The percentage of target species caught is indicated at the top of each radial plot.

The composition of the lures strongly affected the specificity of the catch. Depending on the lure, considerable numbers of Diptera (particularly Muscidae and Tephritidae) and Lepidoptera were caught. We analyzed the specificity of the lures by expressing it as a percentage of the *L. botrana* catches, which demonstrates that specificity as a function of target species decreases with the increasing number of components in the blend (Figs. 5 and 6). The specificity of the lures was also affected by the sampling period. During the first flight period, the complex blend had a very low specificity (2-4% only). This was largely due to a combination of low *L. botrana* populations and relatively high captures of other taxa. Conversely, during the third flight period, the high population of *L. botrana* increased the specificity of all lures. Over the entire season, the 2-component blend was more specific (53-58-96%) than the major compounds (2-64-65%) or the complex blend (4-54-77%), with minor differences detected during the second flight (Fig. 5).

We further assessed specificity as a function of the ratio of the AA/2-PET blend in the third flight. Increasing the ratio of acetic acid led to an increase in the capture rate of *L. botrana* (Fig. 6). Conversely, bycatches, particularly the capture rate of lacewings, increased as a function of 2-PET load in the same blend.

## Discussion

Whereas it is generally agreed that modern agriculture needs a sustainability overhaul, the best trajectory to sustainable production is less clear and progress in sustainable innovation is slow. Today, control of pests and diseases still relies heavily on cover sprays. Innovations are sorely needed that selectively target pests and reduce or eliminate cover sprays, minimising the impact on an already dwindling insect community^2,24^. Odor-based methods offer this perspective through selectively attracting or confusing target insects. Lures laced with attractants, phagostimulants and small amounts of insecticides can selectively target pest species, while their specificity avoids bycatches from the food web. Unfortunately, bycatches are not consistently reported, which makes evaluation of lures in terms of sustainable control of pests difficult. Although lures have been reported for numerous pest insects, they may be broadly attractive and similarly to insecticides, impact non-target species.

In our study, we empirically evaluated the effect of ratio, release rates and composition of a lure^16^ on capture of *L. botrana* and analysed the concurrent effect on specificity. The lures attracted both male and female *L. botrana*, and could be used to support pheromone-based intervention methods. Aiming to further increase the effectiveness of the lure, we found that such increases may come at the expense of specificity of the lure. Increased release rates and blend complexity strongly decreased specificity, while not always increasing attractiveness to *L. botrana*. There thus appears to be a tradeoff between attractiveness and specificity, and the ‘most attractive’ in terms of total catch may not necessarily be the ‘most attractive’ in terms of specificity and thus sustainability.

### A two-component blend alone can selectively attract *L. botrana*

Based on previous work with AA and 2-PET, where considerable numbers of *L. botrana* were caught^16^, we assessed whether the lure’s attractiveness could be further enhanced by changing the release rate and ratio of AA and 2-PET. Both 2-PET and AA appeared to be necessary for capturing *L. botrana*. The two compounds synergize with each other, reminiscent of components in a pheromone blend, where frequently small amounts are necessary and sufficient to increase attraction^25^. However, in another study in Hungary, 2-PET did not synergize with AA^25^, although the authors used a much higher dose of AA (3000 mg instead of 500 mg) and another dispensing technique, making the results hard to compare with our study. It is rather surprising that a lure consisting of so few and such generic fermentation volatiles can be so selective. Acetic acid is a common fermentation volatile and indeed a constituent of lures for diverse insect taxa, including flies, moths, lacewings and wasps^26–31^. 2-PET is another rather general microbial volatile. It indicates the breakdown of phenylalanine and thus a protein source, with similar or derived compounds attracting various insect taxa^25,29,32,33^. That a combination of these two can be selective, may indicate that even though insects commonly rely on fermentation volatiles for adult feeding, the olfactory circuitry of different species key into different components in orientation. This is supported by recent work on tephritid fruit flies^34^, where an ecological niche-driven divergence in the detection of fruit volatiles was measured, in spite of these sources generically being attractive to all species tested.

Besides, differential tuning to fermentation volatiles, the high selectivity of the 2-component blend to *L. botrana* (Fig. 6) may also result from its dominant presence in the vineyard, whereas selectively would be much lower in situations where this is not the case. The fact that in early season catches (1st and 2nd flight) selectivity was dramatically lower, underlines this. Claims about a lure’s selectivity thus need verification throughout the flight season and possibly in different geographical areas.

A higher load of AA increased capture rates of *L. botrana* while higher 2-PET loads increased capture rates of lacewings. This underlines that research should not solely focus on increasing capture rates of the target insect species, but carefully balance ratio, load and composition to reduce bycatches.

### Other fermentation volatiles lacked synergy, and decreased specificity of AA and 2-PET

As a blend consisting of only AA and 2-PET is far removed from a fermentation volatile mimic, we reasoned that addition of other fermenting volatiles could perhaps synergize the 2-component blend. Numerous reports have shown fermentation-based blends with quite different constituents, though often containing AA, ethyl acetate and primary alcohols, as being attractive to other insect taxa^26,35^. Among moth species, leafrollers have received considerable attention with studies on *Archips, Cydia, Pandemis*, *Spilonota*, *Epiphyas* and *Choristoneura* spp.^36,37^. Attractants comprised both constitutive plant compounds such as pear ester and induced volatiles released upon feeding damage by leafroller larvae, such as 2-PET, benzyl alcohol and benzyl cyanide. However, plant volatiles only significantly attracted when combined with AA^36,38^.

Surprisingly, however, in our study none of the fermentation volatiles (as identified in^16^) increased catches of *L. botrana*. A number of reasons could underlie this. As we only tested a single load and ratio, we cannot exclude that other doses and ratios would have induced increased capture rates. Furthermore, the release rates and strong synergistic effect of 2-PET on AA may have obscured additive effects of the additional fermentation volatiles. Finally, the release rates of the compounds from the vials may have differed considerably, something that was not verified in this study. Future work could expand on the current by evaluating these factors.

Although the additional fermentation volatiles did not increase *L. botrana* captures, they did significantly increase attraction of other insect taxa, among which other pests: adding alcohols attracts *Musca* spp.; adding major fermentation compounds attracted Tephritidae in the first flight and *Drosophila* spp. in the last. The highest catches for all species were observed with a complex blend of 13 compounds. We also confirm a synergy between AA and 3-methyl-1-butanol for *Hypsopygia costalis* (Lepidoptera: Pyralidae^28,39^;). Apparently, lures can be designed from generic fermentation volatiles, that, depending on their composition and release rate, can be selective for certain insect taxa. These results only further underline the significance of the synergy between AA and 2-PET for *L. botrana* specifically.

We suggest that future studies such as this one should always carefully analyze blend ratio, composition and release rates to optimize not only attractiveness for the target pest, but also selectivity to avoid non-target species.

## Conclusions

This study demonstrates that in spite of our expectations, lures consisting of only two components were sufficient to capture *L. botrana* males and females. Addition of other microbial volatiles did not enhance attraction. The fact that a very limited number of volatiles can selectively attract certain insect species, offers a perspective that selective lures can be developed, not only based on host-plant specific odors, but can even be derived from generically attractive substrates such as fermentation sources. Such selective lures will greatly support monitoring, by reducing or eliminating the need for identification of catches. In addition, the large numbers caught offer the perspective of the use of such lures in sustainable control, by targeting the damaging sex directly (in e.g. attract & kill), rather than indirectly (such as e.g. mating disruption). Further optimization of attractiveness, specificity, as well as the dispensing technology of the volatile components are needed, while on-going research on volatiles from (induced) hosts and microbial breakdown may provide additional volatile candidates for this.

With moth pheromones as hallmark of attractiveness, chemical ecologists readily focus on finding ‘highly attractive’ lures with a similar potency. However, orientation to feeding and oviposition substrates occurs continually over a moth’s lifetime, in contrast to mate orientation. Accordingly, the probability of contacting moderately attractive food or oviposition lure is arguably much higher, as long as they are well dispersed throughout a crop^40,41^. Selective lures with reasonable attractiveness, such as the one described, may thus be ‘good enough’ from a control perspective, while simultaneously highly desirable from a sustainability perspective.

Finally, in our study, we took great care to analyze not only catches of *L. botrana*, but also bycatches. These show that specificity can readily be compromised by changes in release rates, ratios and composition of lures. Unfortunately, there exists as of yet no strong tradition for optimizing for specificity, or for reporting bycatches. There are neither standardized procedures (how/frequency to sample, season, geographic locations) or statistics (to what taxonomic level, what diversity indexes, etc.) with which to express these bycatches. This hampers comparing results across studies and lures, and obstructs the evaluation of results in terms of sustainability. Yet, reporting on bycatches can accelerate the development of selective lures for other pest species, such as reported here for dipteran and lepidopteran. We suggest that future studies always report on bycatches to accelerate sustainable innovations in pest control.

## Supplementary information

Supplementary information

## Data Availability

All data analysed during this study are included in this published article (and its Supplementary Information files).
